# Elimination of *E. faecalis* with NaOCl versus chlorhexidine gluconate from primary molar root canal systems: an ex vivo model study

**DOI:** 10.1007/s00784-024-05621-6

**Published:** 2024-04-23

**Authors:** Shmueli Aviv, Yaya Alin, Lam Neta, Haim Yael, Zamsky Lada, Fux Noy Avia, Ram Diana, Moskovitz Moti, Polak David

**Affiliations:** 1 Department of Pediatric Dentistry, Faculty of Dental Medicine, Hadassah Medical Center, Hebrew University, P.O. Box 12272, Jerusalem, 91120 Israel; 2 “Bina” Program, Faculty of Dental Medicine, Hebrew University of Jerusalem, Jerusalem, Israel; 3 In private practice, Jerusalem, Israel; 4 Department of Periodontology, Faculty of Dental Medicine, Hadassah Medical Center, Hebrew University, Jerusalem, Israel

**Keywords:** *Enterococcus faecalis*, Root canal treatment, Primary molars, NaOCl, Chlorhexidine gluconate, Dental pulp disease, Primary teeth

## Abstract

**Objectives:**

This ex vivo human study aimed to evaluate the efficacy of NaOCl and chlorhexidine gluconate (CHG) irrigations in eliminating *Enterococcus faecalis* from the RCS of primary molars.

**Materials and methods:**

Disinfected extracted primary molars were inoculated with *E. faecalis* for 24 h. Then, the RCS samples were then irrigated with either 2.5% NaOCl, 0.2% and 2% CHG, or sham saline. The samples were collected immediately after irrigation; and 24 h later, the bacterial viability and counts were measured using blood agar and qRT-PCR, respectively. Histological sections were used to measure *E. faecalis* penetration and viability in dentin tubules using fluorescence microscopy.

**Results:**

The recovery of viable *E. faecalis* after the irrigation of the primary molars showed more significant bactericidal effects of NaOCl and 0.2% and 2% CHG than of saline. Immediately after the irrigation, the NaOCl group showed the greatest reduction in *E. faecalis*; and 24 h later, all the groups had lower viable *E. faecalis* than the saline control. The bacterial penetration was also lowest in the NaOCl group, although there was no difference in bacterial viability in the tubules between the groups.

**Conclusion:**

In primary teeth, NaOCl and CHG showed similar degrees of bacterial elimination efficacy in terms of E.faecalis.

**Clinical relevance:**

Within the limitations of this study, NaOCl and CHG have the similar ability to perform endodontic irrigation of primary ex vivo teeth regarding the elimination of E.faecalis, but NaOCl penetrates dentin tubules better.

**Supplementary Information:**

The online version contains supplementary material available at 10.1007/s00784-024-05621-6.

## Introduction

While pulpotomy is a procedure performed in vital pulp treatments in primary teeth, root canal treatment (RCT) is the primary therapeutic option for primary teeth with irreversible pulpitis or pulp necrosis. It is used to clear the root canal system (RCS) of infected tissue debris, bacteria, and toxins and seal it with an appropriate material [1]. The clinical efficacy of RCT on primary teeth depends on appropriate disinfection of the RCS and proper sealing of the root canals [2, 3]. RCT failure occurs when microorganisms are insufficiently eradicated from the root canal, leading to a persistent infection that prevents healing of the periapical and inter-radicular tissues [2, 4–7].

The complex anatomy of the RCS in primary teeth with rich accessory canals at the root furcation makes mechanical debridement insufficient. Moreover, the organic tissue and bacteria in the dentinal tubules are inaccessible for mechanical cleaning and require further chemical irrigation to clear the infected or necrotic tissue [8]. On the other hand, the use of aggressive irrigation agents may damage the surrounding healthy tissue, as such agents may leak through the periapical foramen or the accessory canals. This leakage may also damage the permanent tooth bud adjacent to the periapical foramen and cause morphological defects and irregularities in the mature tooth [9–12].

It is generally recommended that RCS irrigation systems have four key characteristics: antimicrobial activity, the ability to dissolve organic tissue, effectiveness in disinfecting and cleaning root canals, and non-toxicity to the extra radicular tissues [9, 13]. One effective irrigant for primary tooth disinfection is NaOCl [14, 15] due to its antimicrobial properties and its powerful ability to dissolve organic tissue [2, 7, 14]. However, NaOCl can be toxic and can lead to a hypochlorite accident, which causes acute pain, swelling, redness, facial nerve palsy, and other complications in extraradicular tissue [3, 10]. Such risks led clinicians to search for other materials with less potential for harm and less side effects, especially in children [2, 14].

Chlorhexidine gluconate (CHG) is a broad-spectrum antibacterial molecule that is effective against bacteria in infected root canals [2, 7, 13, 14, 16]. Moreover, it binds to dentine and soft tissues and thus, has a prolonged antibacterial effect [16, 17]. It is also less toxic than NaOCl and does not dissolve organic matter [2, 14, 18]. While both NaOCl and CHG are prevalent root canal irrigants that are superior to saline [2], no data exist on which of these materials is superior [18], especially for primary teeth.

*Enterococcus faecalis* is a gram-positive bacterium that is commonly found in persistent endodontic infections of up to 77% [19–22], possibly due to its evasion and resistance mechanisms [22, 23]. In vitro research by Nara et al. examined the efficacy of 3% NaOCl as a root canal irrigation material in samples contaminated with *E. faecalis*. The results showed growth of the bacteria in over 50% of the samples irrigated with NaOCl [24]. Siqueria et al. examined the efficacy of three NaOCl concentrations that ranged from 1 to 5.25% on *E. faecalis* elimination and found that all the concentrations significantly reduced the number of bacterial cells in the root canal and showed large zones of inhibition of *E. faecalis* growth [25].

This study evaluated and compared the effectiveness of 2.5% NaOCl, 0.2% and 2% CHG, and saline in the elimination of *E. faecalis* from root canals of primary molars. Our hypothesis was that NaOCl is more effective in eliminating bacteria compared with CHG or saline.

## Materials and methods

Primary molar teeth that were extracted due to infection or extensive cavities, with remaining roots of at least two-thirds of the original root length, were collected and preserved in 70% ethanol.

The study protocol was approved by the Institutional Human Subjects Ethics Committee of (0312-16-HMO). All the parents or caregivers have signed informed consent.

### Tooth preparation

The RCS from the crown cavity was accessed with a water-irrigated diamond bur, and the pulp tissue was removed with a 30 K file. The apical foramen was sealed with a flow composite (3 M flow Ultimate, MN, USA). When the tooth crown was damaged, the missing crown surface was replaced with a composite (3 M P60 packable, MN, USA). Then, the teeth were sterilized via autoclaving at 121˚C for 15 min and kept dry until use.

### Bacteria

*E. faecalis* (ATCC V583) was grown overnight in brain heart infusion (BHI) broth at 37 °C under aerobic conditions. The bacterial concentration was adjusted to 1.5 × 10^8^ colony-forming units (CFUs) per milliliter. The bacteria were stained with fluorescein isothiocyanate (FITC), as previously described [26].

### Ex vivo endodontic infection in a primary tooth model

Under sterile conditions, the teeth were staged in a 24-well plate, and the bottom of the plate was coated with sterile orthodontic wax. The RCS was filled with 60 ul of *E. faecalis* in fresh BHI broth (at an optical density of 0.1) for 48-h incubation in anaerobic conditions at 37˚C, to which 20 ul of fresh BHI broth was added after 24 h.

### Endodontic treatment

In the first set of experiments, the teeth were divided into the following 3 groups (*n* = 10 teeth/group): the 2.5% NaOCl group, the 0.2% CHG group, and the saline group. In the second set of experiments, the teeth were divided into 4 groups (*n* = 10 teeth/group; the 2.5% NaOCl group, the 0.2% CHG group, the 2% CHG group, and the saline group).

After the *E. faecalis* biofilm formation, the RCS in each group was washed with a 5-ml irrigation solution. Then, three paper points (each with a size of 25) were placed in each canal for 15 s, collected into tubes with 500-ul phosphate buffered saline (PBS) each, vortexed for 15 s, and seeded on blood agar in eight 10-fold dilutions for CFU calculation.

After the irrigation treatment, the RCSs of all the groups were again filled with fresh BHI broth for an additional 24-h incubation. As before, the RCSs were again collected using a paper point for CFU calculation.

### Microscopic analysis

All the teeth were sectioned using a water-chilled microtome to a width of 0.2 mm and then stained with propidium iodide solution. The analysis was performed under a fluorescent stereo fluorescence microscope (Nikon SNX, Tokyo, Japan). Images were obtained to measure the staining depth of the dentin tubules from the RCS crown cavity walls using ImageJ software.

### qRT-PCR

Bacterial DNA was extracted using a DNA extraction kit (Qiagen, Venlo, the Netherlands). The *E. faecalis* was quantified using SYBR-Green-based quantitative real-time PCR (PCR Biosystems, London, UK) and primer sets of GTTTATGCCGCATGGCATAAGAG (forward) and CCGTCAGGGGACGTTCAG (reverse). All the reactions were carried out in duplicate, and all the plates included a standard curve.

### Statistical analysis

The experiments were conducted in triplicate and repeated twice. The results are expressed as the average of all the replicates with a standard error. Statistical significance was calculated with a one-way ANOVA test and Bonferroni correction. All the fold change analyses were performed using the ANOVA ranks statistical test. Statistical significance was defined as *P* < 0.05, and analysis was performed with SigmaStat 2.01 software.

## Results

**Fig. 1:**
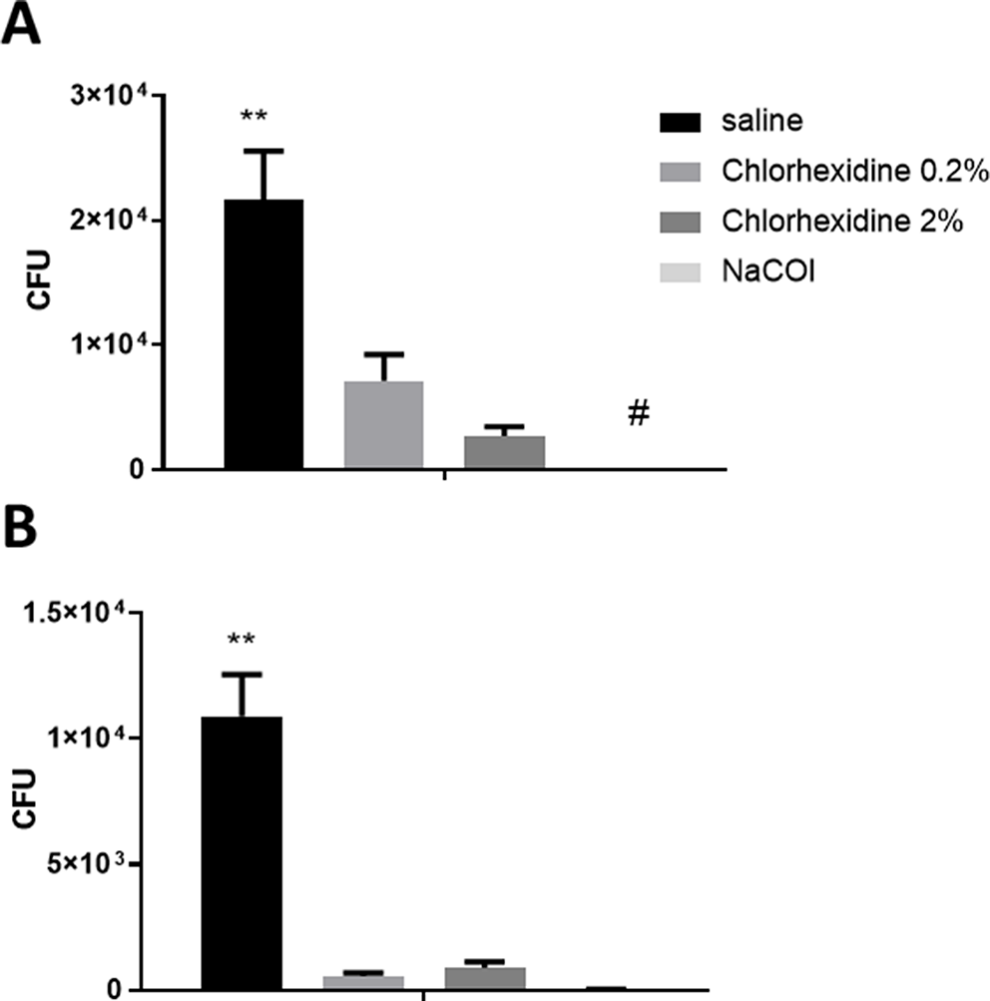
*E. faecalis* recovery from the primary tooth root canal system (RCS) following endodontic irrigation. Primary teeth were infected with *E. faecalis* and then washed with NaOCL (Dakines), chlorhexidine gluconate (CHG) at different concentrations, and saline. The samples from the canals were taken immediately after the irrigation (**A**) and 24 h after the irrigation (**B**) for colony forming units (CFU) calculation. The results are expressed as means and SDs. ** indicates a statistical difference between the control and all test groups. # indicates a statistical difference between the test groups

The first set of experiments tested the recovery of viable *E. faecalis* following endodontic irrigation of primary molars. The results show more significant bactericidal effects of NaOCl and 0.2% and 2% CHG than of saline (Fig. 1). Immediately after the irrigation, the NaOCl group showed the greatest reduction in *E. faecalis* (Fig. 1a). After 24 h of irrigation, all the groups had lower viable *E. faecalis* than the saline group (Fig. 1b).

**Fig. 2:**
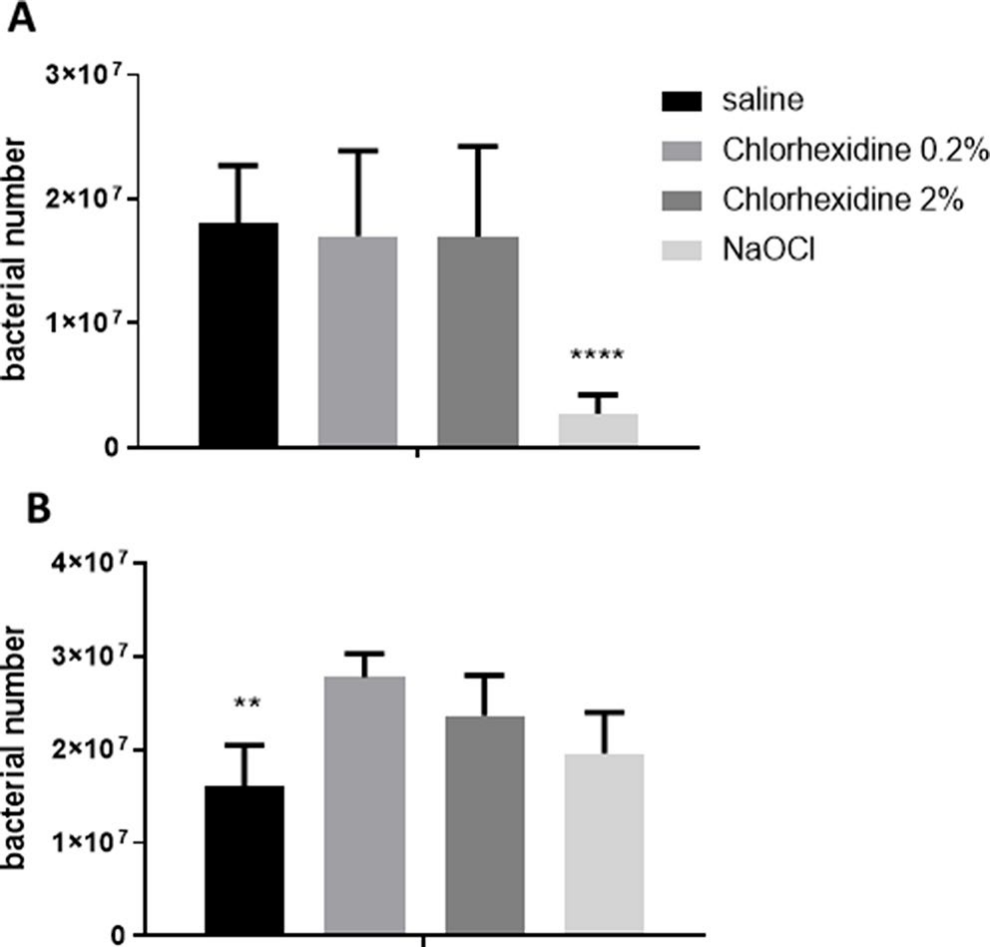
*E. faecalis* counts from the primary tooth RCS following endodontic irrigation. Primary teeth were infected with *E. faecalis* and then washed with NaOCL (Dakines), CHG at different concentrations, and saline. The samples from the canals were taken immediately after the irrigation (**A**) and 24 h after the irrigation (**B**) to measure the bacterial levels using qPCR. The results are expressed as means and SDs. ** indicates a statistical difference between the control and all test groups (*P* < 0.01). **** indicates a statistical difference between the control and all test groups (*P* < 0.001)

Since in endodontic pathology, bacterial debris can trigger a host response that leads to tissue damage, we next examined the bacteria that remained after the irrigation, regardless of their viability, by quantifying the *E. faecalis* DNA presence in the RCSs. The results showed that immediately after the irrigation, only NaOCl was able to reduce the bacterial DNA (Fig. 2a). However, 24 h after the irrigation, all the test groups harbored similar levels of bacterial DNA (Fig. 2b).

**Fig. 3:**
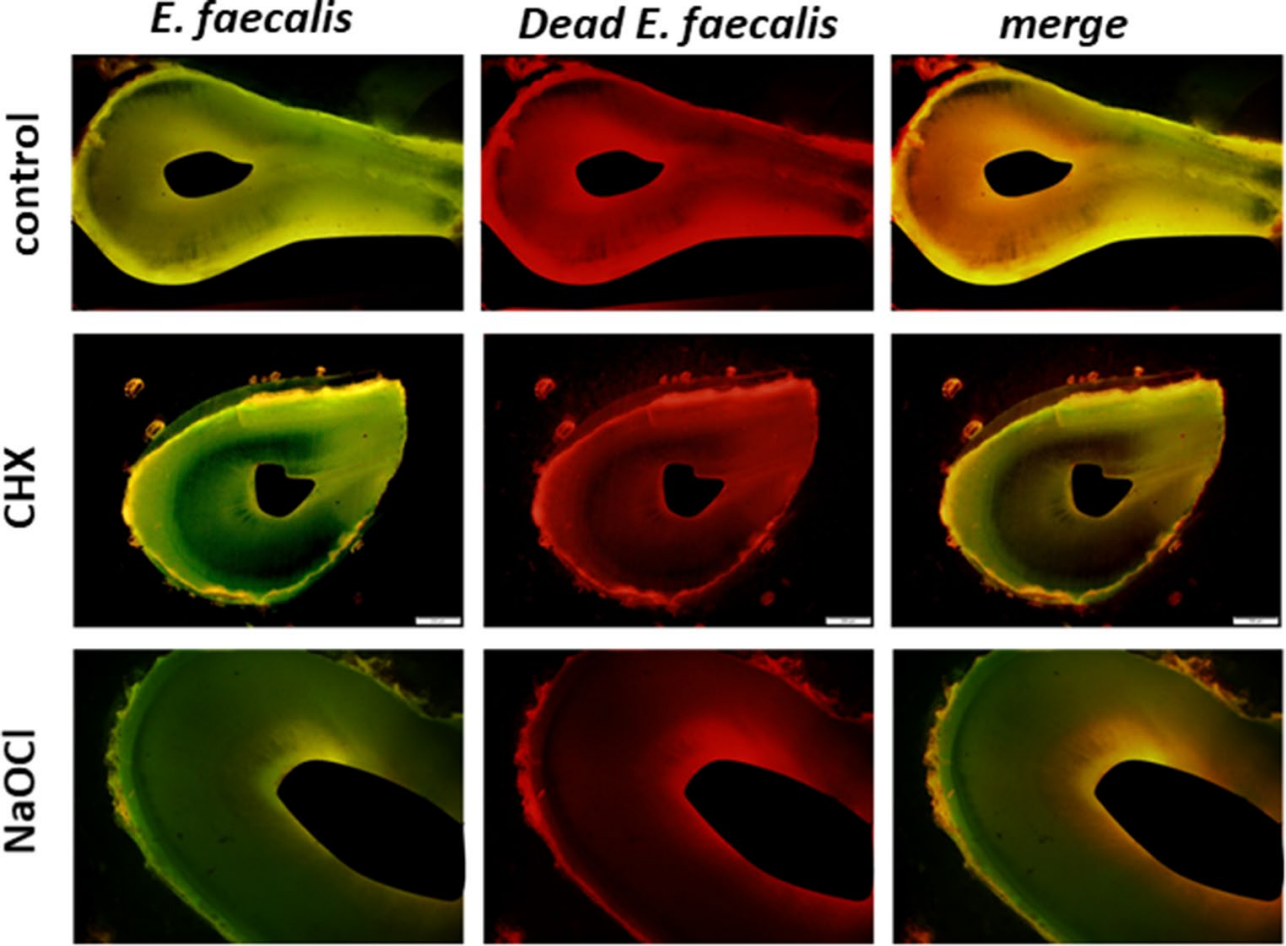
Microscopic images of primary teeth infected with *E. faecalis* and following endodontic irrigation. Primary teeth were infected with fluorescein isothiocyanate (FITC)-tagged *E. faecalis* and then washed with NaOCL (Dakines), CHG at different concentrations, and saline. Then, the teeth were sliced into 1-µm-thick strips and stained with propidium iodide. The images depict representative strips stained for the bacteria (green) or dead bacteria (red) and their merging

**Fig. 4:**
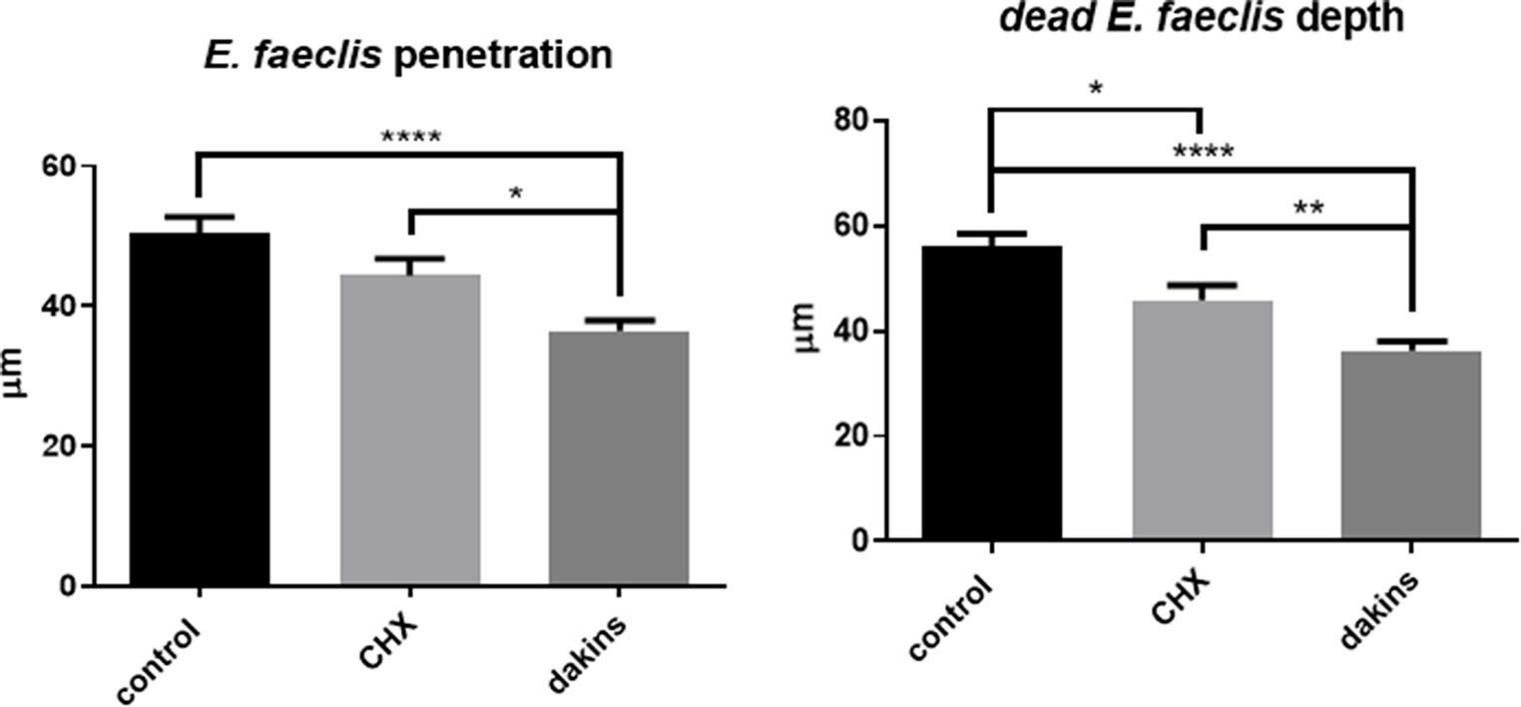
*E. faecalis* penetration and viability into dentin tubuli following endodontic irrigation. Primary teeth were infected with FITC-tagged *E. faecalis* and then washed with NaOCL (Dakines), CHG at different concentrations, and saline. Then, the teeth were sliced into 1-µm-thick strips and stained with propidium iodide. The distance from the inner border of the RCS to the tubuli of the *E. faecalis* (FITC-positive bacteria) or the dead *E. faecalis* (propidium iodide (PI) and FITC-positive bacteria) at 10 sites/slide was measured. The results are expressed as means and SDs. ** indicates a statistical difference between the control and all test groups (*P* < 0.05). ** indicates a statistical difference between the control and all test groups (*P* < 0.01). **** indicates a statistical difference between the control and all test groups (*P* < 0.001)

Next, slides were used to create a visual representation of the bacterial penetration into the dentin tubules during the experiment. The results show that in all the groups, *E. faecalis* penetrated the dentin tubules from the RCS cavity (Fig. 3). The quantification of the depth of the bacterial penetration and the bacterial viability showed that in the NaOCl group, the depth of the bacterial penetration was the lowest, followed by medium penetration in the CHG groups and the highest penetration in the saline group (Fig. 4a); and the same pattern was seen for the bacterial viability (as propidium iodide (PI) measurement), with the NaOCl group showing the lowest penetration depth, followed by medium penetration in the CHG groups, and the highest penetration in the saline group (Fig. 4b).

## Discussion

This study demonstrated that NaOCl and CHG have a similar ability to perform endodontic irrigation of primary ex vivo teeth in terms of E.faecalis eliminiation, but that NaOCl penetrates dentin tubules better. This rejects the study original null hypothesis that claimed that NaOCl would show superior efficacy to CHG, Nonetheless, the results show that at early stage (immediately after irrigation) the NaCOl did show superiority to the other tested groups. At all tested times both NaCOl and CHG were superior to the negative saline control.

The risk of damage to the permanent tooth bud is especially high in children. Dental treatment of children is challenging in any case, but the use of toxic agents, together with management of the child’s behaviors, carries the risk of an NaOCl accident occurring due to leakage of the irrigant to the interradicular area, which may ultimately damage the permanent tooth bud [3, 10].

As bacteria and their byproducts initiate endodontic inflammation [1], RCT aims to reduce bacterial load through disinfection. However, the efficiency of mechanical disinfection methods in RCT on deciduous teeth is limited due to anatomical intricacies, curved canals, wide apical foramina, accessory canals in the floor of the pulp chamber, and the presence of a permanent tooth bud below the treated tooth. Furthermore, bacterial biofilm can reside in dentinal tubules at a depth of 500–1,000 μm [27]. Indeed, the keystone endodontic pathogen *E. faecalis* [28–30] is difficult to remove from root canals because it penetrates dentinal tubules. This conforms to the fact that *E. faecalis* is responsible for approximately 80–90% of RCT failure [23, 31]. This is compounded by the fact that *E. faecalis* flourishes in alkaline surroundings comparable to those encountered in calcium hydroxide root canal dressings and thus, can grow and multiply in the presence of calcium hydroxide [20, 21, 23, 32]. Therefore, irrigation is an essential step in RCT. The most common irrigation materials are NaCOl and CHG [2, 7, 9, 13, 33]. However, none of these two materials is considered superior to the other [34–36]. Their optimal concentrations for *E. faecalis* elimination are 5.25% for NaOCl and 2% for CHG [25, 36]. Current data show similar bactericidal effects of 2.5% NaOCl and 2% CHG in diminishing the proliferative capacity of bacteria. Nevertheless, while this effect was immediate with NaOCl irrigation, CHG showed the same effect only 24 h later. Moreover, unlike NaOCl, lower concentrations of CHG still have bacteriostatic effects [2, 17] but cannot dissolve organic tissue [17], which is a key advantage of NaOCl. Another advantage of NaOCl is its ability to penetrate dentinal tubules to a depth of approximately 300 μm [27]. In this study, the saline control group showed the deepest penetration of bacteria in the dentin tubule, followed by the CHG group and the NaOCl group. These results suggest that NaOCl reaches deeper into the tubules than CHG or saline. Since deeper penetration allows for better irrigation and clearing of the substrate for dead bacteria staining, the presence of dead bacteria was proportionally lower in the NaOCl group. Nevertheless, despite the high penetration of NaOCl, the rates of dead bacteria in this group were similar to those of the other groups.

In any case, as *E. faecalis* can penetrate tubuli to as deep as 500–1,000 μm, it may not be fully eliminated by bacteria elimination methods [37].

These results raise the question of whether the disinfection endpoint of irrigation procedures is caused by the bactericidal qualities of the material itself or is also heavily influenced by the actual irrigation procedure. These findings highlight the importance of canal irrigation during RCT.

## Conclusions

Both 2.5% NaOCl and 2% CHG were effective in eliminating *E. faecalis* from the root canals of primary molars, although NaOCl had slightly better results.

Since this is an ex vivo study, further research is needed to assess the abilities of root canal irrigants to properly disinfect the RCS in primary teeth.

### Study limitations

This is an ex-vivo study, which does not fully imitate the natural environment in the clinic (such as immune system response, humidity the effects of irrigation material leakage, bacterial composition, and biofilm structure). Furthermore, visual access to the canal orifices is different in the laboratory in comparison to the clinical situation. Another aspect that is different in endodontic treatment in primary teeth and is not reflected in our work, is the root canal dressing material that in contrast to RCT in a permanent tooth, also adds an antibacterial effect.

### Electronic supplementary material

Below is the link to the electronic supplementary material.

Supplementary Material 1
